# Perspectives of stakeholders of the free maternity services for mothers in western Kenya: lessons for universal health coverage

**DOI:** 10.1186/s12913-022-07632-z

**Published:** 2022-02-19

**Authors:** Beverly Marion Ochieng, Margaret Kaseje, Dan Clement Owino Kaseje, Kevin Oria, Monica Magadi

**Affiliations:** 1 Tropical Institute of Community Health (TICH), P.O. Box 4074-40103, Kisumu, Kenya; 2 University of Hull, , Cottingham Road, Hull, HU6 7RX UK

**Keywords:** Universal Health Coverage, Maternity, Skilled Delivery, Ante Natal Care, Post-Natal Care, Quality Care, Cost Effectiveness, Social Protection

## Abstract

**Background:**

The strategic aim of universal health coverage (UHC) is to ensure that everyone can use health services they need without risk of financial hardship. *Linda Mama* (Taking care of the mother) initiative focuses on the most vulnerable women, newborns and infants in offering free health services. Financial risk protection is one element in the package of measures that provides overall social protection, as well as protection against severe financial difficulties in the event of pregnancy, childbirth, neonatal and perinatal health care for mothers and their children.

**Purpose:**

The aim of this study was to find out the extent of awareness, and involvement among managers, service providers and consumers of *Linda mama* supported services and benefits of the initiative from the perspectives of consumers, providers and managers.

**Methods:**

We carried out cross sectional study in four sub counties in western Kenya: Rachuonyo East, Nyando, Nyakach, and Alego Usonga. We used qualitative techniques to collect data from purposively selected *Linda Mama* project implementors, managers, service providers and service consumers. We used key informant interview guides to collect data from a total of thirty six managers, nine from each Sub -County and focus group discussion tools to collect data from sixteen groups of service consumers attending either antenatal or post-natal clinics, four from each sub county, selecting two groups from antenatal and two from postnatal clinics in each sub county. Data analysis was based on thematic content analysis.

**Findings:**

Managers and service providers were well aware of the initiative and were involved in it. Participation in *Linda Mama*, either in providing or using, seemed to be more prominent among managers and service providers. Routine household visits by community health volunteers to sensitize mothers and community engagement was core to the initiative. The managers and providers of services displayed profound awareness of how requiring identification cards and telephone numbers had the potential to undermine equity by excluding those in greater need of care such as under-age pregnant adolescents. Maternity and mother child health services improved as a result of the funds received by health facilities. *Linda Mama* reimbursements helped to purchase drug and reduced workload in the facility by hiring extra hands.

**Conclusion:**

The initiative seems to have influenced attitudes on health facility delivery through: Partnership among key stakeholders and highlighting the need for enhanced partnership with the communities. It enhanced the capacity of health facilities to deliver high quality comprehensive, essential care package and easing economic burden.

## Background

The strategic aim of universal health coverage (UHC) is to ensure that everyone can use the health services they need without risk of financial impoverishment, no matter what their socio-economic situation. The over-arching concept of UHC takes a broad view of the services that are needed for good health and well-being. *Linda mama* (taking care of the mother) initiative focuses on women, new-borns and infants in offering free health services. Financial risk protection is one element in the package of measures that provides overall social protection, as well as protection against severe financial difficulties in the event of pregnancy, childbirth, neonatal and perinatal health care for mothers and their children.

Kenya is far from achieving the health related sustainable development goal (SDG) as defined by the Inter-Agency and Expert Group on Sustainable Development Goal Indicators (E/CN.3/2016/2/Rev.1), in United Nations Development Programme (UNDP) [1], focusing on ensuring healthy lives and promoting well-being for all ages by 2030, measured by reduction of the global maternal mortality ratio to less than 70 per 100,000 live births**,** while Kenyan rate is estimated at 510 by the World Bank in 2015 [2]; reduction of neonatal mortality to 12 per 1,000 live births while Kenyan neonatal mortality rate continues to be twice higher, at 21 per 1000 live births [3]. These Kenyan indicators underline the critical importance of *Linda Mama* initiative, in order to contribute to achieving UHC towards Sustainable Development Goal (SDG) 3. Kenya has a 58% coverage of four or more antenatal care (ANC) visits, 62% of births are attended by a skilled healthcare provider and 43% of mothers do not receive skilled postnatal care (PNC) within the first six weeks after delivery. (Kenya demographic and health survey [4]. In Homa Bay (60.4%), Kisumu (69.2%) and Siaya (70.4) of women were assisted by a skilled service provider during delivery [4]

The sources of health financing in Kenya includes tax revenue, out of pocket payments, donor and insurance accounting for 37%, 26.1, 23.4% and 13.5% of the total health expenditure respectively [5]. A considerable proportion of households in Kenya (7.1%) experienced catastrophic health expenditures in 2018 translating into one million Kenyans being pushed into poverty [6]. User fees as a financing mechanism has been reported to limit access to services and leads to increased inequities [7–9]. A systematic review by [10] reported that removal of user fees in low-income and middle-income countries results in increase in ANC visits and facility deliveries. There is strong evidence that user fee removal is associated with an increase in facility deliveries and a decrease in neonatal mortality [10, 11].

As early as 2004, Kenya Government abolished user fees in all primary level facilities except a registration fee of Kenya Shillings 10 (USD 0.33) and KES20 (USD 0.67) at dispensaries and health centers, respectively [12]. The same authors [12] reported low compliance with the 10/20 policy as health facilities still charged patients higher fees [13]. As a result the Kenya Government repealed the 10/20 policy in 2013 and introduced free maternity policy in all public healthcare facilities [14], and in order to improve compliance with the free maternity policy the national government compensated health facilities for the lost revenue. The free maternity policy improved utilisation of services, though, according to [15], swveral challenges persisted.

In an effort to tackle some of these challenges the Government of Kenya moved the management of the free maternity policy from the Ministry of Health to the National Health Insurance Fund (NHIF) and expanded service access to include private providers. The revised free maternity policy labelled *“Linda Mama”* [16] is one of Kenya's pro-poor policies intended to benefit the poor and vulnerable, by reducing inequities in access to maternity services. The *“Linda mama”* initiative seeks to cover all pregnant women, mothers and their newborns to ensure they have access to the services they need, regardless of their economic status, and thus ensure comprehensive financial risk protection for the poor during pregnancy and delivery. Evidence suggests that while this policy was intended to cover ANC, deliveries and PNC, in practice only deliveries were covered [17].

### The service package

Phase 1 of the programme started in April 2017 when NHIF contracted private,for-profit and faith-based healthcare facilities to offer delivery services. Phase 2 began in July 2017, when NHIF contracted all public healthcare facilities to offer delivery services. Phase 3 was rolled out in March 2018. It included expansion of the benefit package to include ANC and PNC care across contracted public, private and faith-based healthcare facilities [18].

*Linda mama* had a range of an integrated comprehensive service package available to the pregnant women and their children free of charge at the time of need. The package included: Antenatal care (A*NC),* maternity, skilled delivery care, post-natal care (PNC), basic obstetric emergency care as well as referral to comprehensive obstetric emergency care to address complications; child welfare such as Immunization, out-patient clinical services, basic laboratory services various tests were carried out free e.g. HIV, urinalysis, hemoglobin, and clinical treatment on an out-patient or in-patient basis thus covering cost of all sickness expenses.

Mothers and children were treated free, and received other health services during pregnancy, delivery and after delivery free of charge. All this in addition to a full mother package of incentives, which consumers considered “very helpful”. Those who had delivered were given sanitary towels, baby washing basin, clothing, toiletries, baby socks, slippers, diapers, soap, cotton wool, and wipes. They were also given the mother child booklet for free.

### Partnership

The *Linda Mama* initiative was a partnership led and spearheaded by the National Kenya Government, to promote universal health coverage (UHC), the Ministry of Health, and National coordination National Health Insurance Fund (NHIF). Different counties had other partners such as Afya-Halisi, KMET, AMREF and Matibabu, particularly in Siaya, were mentioned as supporting the government’s initiative, in various specific aspects. The Kenya Government and County governments were involved in the provision of staff for ANC, PNC etc. NHIF was involved in reimbursement of funds, and MOH ensured effective service delivery. In Siaya, Afya Halisi was involved in capacity building of service providers, provision of equipment, observation machines, chairs, tables and registers; Ngima for Sure and AMREF were involved in Ultrasound-capacity development and equipment while Matibabu Foundation and comprehensive abortion care (CAC), supported other aspects.

### The study sought to:

1. Find out the extent of awareness, and involvement among managers, service providers and consumers of *Linda Mama*” supported services
2. Describe the benefits of the initiative from the perspectives of consumers, providers and managers
3. Describe the impact of the initiative on attitudes towards skilled health facility delivery.
4. Describe the main challenges and emerging lessons
5. Make recommendations for its improvement and applicability to UHC

## Methods

We carried out cross sectional study in four sub counties in western Kenya: Rachuonyo East, Homabay County, Nyando and Nyakach, Kisumu County, and Alego Usonga in Siaya County (See Table 1). We used qualitative techniques to collect data from purposively selected *Linda Mama* project implementers, managers, service providers and groups of service consumers, applying mainly a narrative study design as described by Butina [19]. The data we collected aimed to reveal the experiences of managers, service providers and consumers, with regard to their engagement and interactions with the *Linda mama* initiative. The information obtained was to give insight into their views, perspectives and beliefs concerning involvement with the initiative in order to learn its achievements, what has worked or not worked and challenges, as well as the lessons that can be learnt from their experiences to inform the contribution of the initiative to universal health coverage. We used key informant interview guides to collect data from a total of thirty six managers nine from each Sub -County and focus group discussion tools to collect data from sixteen groups of service consumers attending either antenatal or post-natal clinics, four from each sub county, selecting two groups from antenatal and two from postnatal clinics in each sub county.. These were considered knowledge rich as they would have had adequate exposure to the initiative and decided to register or not to register and hence had a story to tell about it.

**Table 1:** Study sites

	**County**	**Sub County**
1	Homa Bay	Rachuonyo East
2	Kisumu	Nyando Nyakach,
3	Siaya County	Alego Usonga

All participants provided written informed consent to participate in the study. To ensure high quality of data, the Principal Investigator (PI) and study coordinators supervised data collection, and reviewed issues arising in the field at daily evening meetings to ensure that each study team followed and complied with the protocol. The study protocols were reviewed and approved by Great Lakes University of Kisumu Ethical Review Board, The study was carried out by Tropical Institute of Community Health a department under the faculty of health sciences in the University.

### Data analysis

Qualitative data obtained were coded and synthesized into emerging themes and subthemes from the views expressed by the participants, guided by a coding frame, based on objectives of the study, and interview guides to synthesize data into coherent groups of similar statements, to generate information required for assessing progress made by the initiative against UHC, and SDG indicators, based on thematic content analysis as described by Miles and Huberman [20].The synthesized information was then examined and interpreted leading to specific conclusions, emerging from the data. Quotes of what the participants said word-for word was inserted to illustrate specific conclusions, emerging from the data as well as challenges, lessons and recommendations to improve the uptake of the initiative the maternal health goal of the Kenya Government. We developed qualitative analysis and coding frameworks based on the themes presented in the specific research questions and categories of respondents in the protocol.The following stages were followed in the analysis of the qualitative data. Transcribed interviews were analyzed through thematic qualitative analysis using the NVivo qualitative analysis software package version 12. The coding process identified similarities, outliers, and recurring themes in interviews. We created charts and analysis matrixes using headings from the thematic framework. Headings and emerging sub themes were added as suggested by the data during analysis.

## Results

### Awareness, involvement, engagement with *Linda Mama* (taking care of the mother)

All managers and service providers interviewed (36) in all three counties, Homabay, Kisumu and Siaya, were involved in the *Linda Mama* initiative, but only a small fraction of consumers, among ante-natal attendees had heard of the initiative and fewer still had actually enrolled. Post-natal mothers were more aware and more of them had registered. There appeared to be a knowledge and awareness gap among those that had not reached the health facility. The majority of post-natal consumers in Homabay 11 out of 14 had heard of *Linda Mama* initiative, while only 3 out of 14 antenatal respondents had heard of it. Overall, of 20 post-natal respondents only three had not registered, the majority 17 had registered. This was similar in Kisumu County where 19/22 had heard of *Linda Mama* and the same number 19/22 had registered. However, in Siaya, less than half the respondents 4/11, had heard of the initiative and had registered.

### Recruitment

In all counties, according to the respondent consumers who had come for clinics, they had been recruited to access *Linda Mama* services during the first ANC visit and were registered. A woman had to be pregnant to register. All mothers who came in and were pregnant were eligible. They needed a photocopy of the first page of their identification (ID) card, was photocopied and filed, In the absence of own ID card they could use the ID of spouse or mother. Adolescents without IDs carried their guardians’ ID. Some of the women were registered through the community health volunteers (CHV) who referred them to ANC and other services then they were registered. For some without IDs, they used ANC numbers.

Kisumu providers reported the use of phone numbers (self or spouse or mother). They also used mobile phones /smart phones, applying a system in which they would dial (*263#) on Safaricom and it would bring options leading to registration. The community health volunteers (CHVs) could also register the women using their mothers’ mobile phone by dialing (*263#) then referring them to the facility. Registration could also occur at health facilities during any visit or even during delivery when the mother had not been registered in any facility. After registration mothers were given an m-number at the health facility. That notification number was written in their ANC booklet, and would enable them to obtain maternity services and post natal care anywhere in Kenya. Phone numbers were required for follow-up of mothers by the facility to reduce ANC defaulters. They were formally admitted in the maternity or postnatal register.

“Once a mother has M- number then she can deliver anywhere in Kenya” Explained a manager, Kisumu County.

Dissemination of *Linda Mama* information was reinforced through community awareness, opinion leaders, service providers informing mothers, and clients also informing one another, telling them to visit the facility.

Some of the factors that influenced positive outcomes in the *Linda Mama* initiative were *“Health talk during visit, sensitization to community by CHVs, maternity and ANC open days and increased partners involvement”* said a health manager, Homa Bay County.

“When expectant mothers visit hospital, there is a staff in charge of registering them, registration is free, there are two nurses doing the registration”. Explained a service provider from Homabay.

“Mothers are recruited at the first ANC visit and registered into the Linda Mama programme for free” Said a health manager Siaya County.

## Benefits of the initiative

According to the consumers the majority of the benefits were realized during delivery, although they started to be realized as soon as the clients were registered.

“You are treated free of charge, you are given good drugs free even when you are old and for children up to 6 months, we don’t pay for services received by ourselves and our children” explained discussants from Kisumu county.

Another consumer said “It has not helped me at the moment but I hope to get help from it during delivery, I will also get free health services up to 6 weeks of delivery” explained Homabay antenatal discussant.”

“It eases life for mothers particularly for those who do not have money at the time of delivery, they can still just go and deliver at the health facility” explained Siaya discussants.

According to some respondents even the rate of abortions had gone down and explained.

“May be a mother do not have money to deliver in the hospital and so she thinks of aborting but because of this program she will keep the pregnancy because delivery is free, and the care of newborns and infants up to 6 months are free” a discussant clarified.

Services were accessed by more clients by reducing the cost of seeking care to a minimum at the time of need. Free services encouraged mothers to seek services in time. Free ANC resulted in high turn up of mothers, improved general health of mothers and babies, increased knowledge level for mothers as they attended education during clinics, at the health facility.

“Those who were delivering at home are now coming to the facility, before people just used to come for the first ANC visit and disappear, but now it has increased skilled delivery” Siaya service provider explained.

“It is very effective especially for mothers as they are able to get free services like ultra sound,CS and even in patient services for mothers during pregnancy and the child up to the expiry date of the card” service provider Siaya County.

“High turn up of mothers for health services, Improved general health to mothers and babies and Increased knowledge level for mothers as they attend education during clinics” Expressed health manager Siaya County.

“Linda mama initiative has really improved acess to service for HIV positive mothers they get free services like health facility delivery and ANC but there are very HIV negative mothers in the programme (-)-few staff in the linda mother initiative” Said a Health service provider Homa bay county.

It is to be noted that dispensaries were not as involved in *Linda Mama* initiative as higher levels of care.

“Mothers hardly come here for deliveries because it’s a dispensary and we don’t work at night, the effect is felt in bigger facilities” claimed a dispensary respondent from Siaya County.

According to the managers, *Linda Mama* initiative had improved health seeking behavior in terms of improved ANC attendance four visits or more, increased health facility skilled delivery, as the population of mothers delivering in the hospital shot up because of the initiative. There was increased PNC attendance, and Family Planning and other services as well such as immunizations, given at birth, (BCG and birth polio), because of the non-payment by users at the point of use. Complications identified at any stage of pregnancy, during ANC, at admissions for delivery, and peri-natal period were all taken care of free of charge, which was a great relief to users. According to some respondent managers, it covered mothers and their children up to 2 years but after that all children under five years of age received free treatment in public health facilities. They stressed that the initiative had greatly reduced home deliveries, complications, and hence reduced peri-natal morbidity and maternal mortality as well as neonatal morbidity and mortality.

According to service providers “Linda Mama is very effective especially for mothers as they are able to get free services like ultra sound,CS and even in patient services for mothers during pregnancy and the child up to the expiry date of the card” service provider Siaya County.

“Malaria in pregnancy has improved because we give them nets at early stage. They go to term pregnancy because cases of Malaria are reduced. In addition the CHVs get supported by the facility in their activities e.g. community dialogue and action days” explained a service provider from Homabay.

Maternity and MCH services improved as a result of the funds received by health facilities for reimbursements.

“The money for Linda Mama helped to purchase drugs, reduced work load in the facility, improved quality of care to mothers, and are able to get more information regarding their health and that of their babies, and we use part of the funds to renovate our hospital”, explained a hospital based service providers from Kisumu County.

Managers and service providers brought out the fact that the benefit of *Linda Mama* initiative was all round, both health facility and the clients benefitted at the same time. In this way the initiative had improved the quality of service provision, by empowering the facilities in service availability, by making financial resources available to take care of health facility service provision needs. It had thus improved service readiness (stocking of pharmaceutical and non pharmaceutical items, the required infrastructure, the range of services provided, and quality of services and hence service delivery outcomes.

“It is good people accept because it is free it has increased number of clients seeking care better as compared to UHC, Linda Mama is better, the money reimbursed helps in improving the facility, supply of commodities” remarked a respondent manager from Kisumu county.

“There is high turn up of mothers for health services, Improved general health to mothers and babies and Increased knowledge level for mothers as they attend education during clinics” Expressed health manager Siaya County.

It had improved quality of care for the mother and baby, by reducing the cost of referring mothers for specialized services such as ultra-sound services.

“It has a great impact to provision of services. It has enabled acquisition of equipment. It has improved infrastructure in the facility. Part of the Linda mama funds have been used for renovations, repairs, electricity and hiring of a security firm in the facility” explained a satisfied Kisumu service provider.

Consumers testified that the initiative had helped them a great deal “it is totally free**,** the only requirement needed is a photocopy of the ID**,** in addition, there were incentives given after delivery such as pampers, basin, socks, sanitary towels and soap” explained post-natal discussants from Homabay county.

“It helped me when I was to deliver I did not pay any bill at all” explained Kisumu discussants.

Siaya respondents added their voice “It has not helped me yet because I have just registered but I know delivery will be free”.

### Impact on health facility delivery attitudes

The initiative seems to have influenced the attitudes and practices of women with regard to health facility delivery through four pathways that have emerged from our findings that should be considered in the implementation of Universal Health Coverage (UHC).

#### Policy and information dissemination

It appears that clear policy guidelines were disseminated and explained to service providers and managers and were also accessible in the NHIF website for consultation. Sensitization was done well. The *Linda mama* benefits were disseminated to mothers during visits, at maternity, and at ANC and maternity open days. Mothers benefitted also from baby packages, group ANC- porridge given to the mothers. Most of the women delivered at the hospital through the m-card.

“As when I was delivering I stayed in the hospital for 4 days after delivery and we were about 20 mothers who delivered and all had Linda mama cards” Kisumu county, consumer testified.

#### Enhanced health facility capacity

To handle the patients’ needs had regained confidence among consumers on the services at health facilities. From the revenue generated the health facilities were able to pay their bills to suppliers and hire additional staff as they needed for improving access to timely quality of services in terms of structure, process and outcomes.. While increasing the volume of customers served the initiative had reduced workload for service providers, as nurses were able to work in shifts. It was very effective especially for mothers as they were able to get a range of critical services like ultra sound, caesarean section, and even in patient services during pregnancy and the childbirth up to the expiry date of the card, free. Consequently the consumers were convinced that delivery at the health facility was much safer than the alternative providers as complications arising during delivery would be effectively handled.

There was substantial quality improvement, environment was good, equipment, doctors and essential commodities were available, at secondary referral hospitals. Consumers added their testimonies.

“We no longer go to the TBAs, because we are afraid of the complications which might occur during delivery e.g. over bleeding. Moreover, delivery at the health facility is totally free as compared to other places like TBAs. Even less fortunate mothers are able to access care at the health facility. In case a mother is HIV + ve, the TBA cannot prevent the child from being infected hence but health facility does, and is best in my option” Explained Homabay discussants.

Hence it would appear the attitude of consumers had changed, they preferred hospital because it was safe and could solve problems for mothers and for the babies when they arose.

“Mothers now love to deliver at the hospital because the services are good” expressed Kisumu county consumer.

#### Eased family economic burden on health

The program reduced costs for the consumer to the minimum, while not undermining their autonomy and choice for the service provider, where to seek care and to deliver. It lifted the economic burden of care from the consumers. This motivated mothers to seek services and enabled early referrals. In addition the consumers enjoyed incentives of mother-baby packages (basin, slippers, soda, napkins, pads, cotton, tissue, soap, baby shawl and a mosquitoe net). Mothers were able to get tea, porridge and meals after delivery.

“Mothers are given adequate well cooked food, beverages, hot bathing water which make them come in large numbers to deliver here, home delivery is almost nil” according to a Kisumu service provider.

“From January up to now (March) only one home delivery has been reported to the facility, brought by CHV after home delivery” explained a service provider from Homabay.

Similarly, Siaya respondents echoed similar sentiments that the scheme had had a huge impact, because of availability of services free.

“We are having more clients, those who were delivering at home are now coming to the hospital, mothers are motivated to deliver at our facility, they are coming all the way from far because of free, friendly services, mothers no longer come with old “leso” (fabric) to the facility because they will be given a baby shawl” explained a service provider from Siaya.

“We don’t pay anything when we come to deliver, many mothers are delivering in hospital because it is free, as compared to before when most mothers were afraid of the bills” explained Kisumu focus group discussants.

#### Community engagement

The routine household visits by the CHVs to sensitize households, and output-based incentives for referring mothers, seemed to have led to good performance in specific villages where the CHVs are active. They sensitized the community about mother and baby packages and linked households with health facilities. It worked positively both for the facility and the community. However, Siaya respondents stressed the fact that so many mothers still did not know about the *Linda Mama* scheme.

### Main challenges and emerging lessons

Majority of the consumers reported few challenges with the scheme. The main ones included: The requirements of the national identification number (ID) in the registration process had the potential to affect access to care among those most in need. This risk was recognized and some alternatives were introduced to cover everyone, such as young people without ID, so that no one would be excluded. A lingering issue was that mothers were asked to pay for photocopying which introduced a cost to consumer, potentially a barrier to accessing services at the time of need.

“Most young people don’t have IDs so registration can be a challenge and Some mothers don’t have ID cards.” Explained a service provider Siaya County.

“The programme requires mothers to have an ID and a phone and some do not have phones” said service provider Kisumu County.

“Knowledge gap among mothers regarding the initiatives is a real issue and The issue of photocopying documents is a challenge especially when the clients are required to do the photocopy, it is not fruitful, and they end up not bringing the document at the right time, hence missed claims”. Service provider Homa Bay County.

According to the managers and service providers the main challenge was the delay in disbursement of funds to the facilities. The process of claiming was described as tedious. Refund period was not consistent and took too long because the money was channeled through the county government, and processing took too long. It made transport for monthly report submission to the NHIF problematic. The delay affected the motivation among providers and managers.

“Right now, in March, we are still waiting for reimbursement for December, January and February” explained a manger, Kisumu County.

“Refund period is not consistent takes longer and the programme has led to increased workload in the periphery hospitals/facilities”Expained a manager Siaya County.

“Late payment from the initiative, the requirements needed were not clearer in the beginning and even processing the documents.” Health Manager Homa Bay County.

They explained that delayed reimbursement affected work as often they would not have money to buy bundles for internet access, needed for registration and other data processing activities. Service providers complained of a mismatch between service demand and available resources to provide services, many respondents mentioned increased workload.

“It’s quite involving and requires additional staff for effective data capture on a daily basis” explained Homabay service provider..

The challenges that we face in Homa Bay is lack of stable supply of electricity, few staff and a lot of work,it needs someone who is trained, devolving the initiative to the sub-county- everything is done at the county-claiming fee and the payments take time from the National Government. Explained a manager, Homa Bay County.

“Doing photocopying of ID and registration documents involve a lot of work and the maternity services such as delivery beds were often too few for the number of deliveries being conducted” explained Kisumu service providers.

Some mentioned the importance of having a photocopier within the health facility.

“Photocopying registration form outside the facility is tiresome and could lead to some mothers being lost or having delayed access to care” interjected a Siaya service provider.

They noted that the online procedures during registration and other visits required computers and access to internet, as well as constant availability of bundles, affected by reimbursement delays. Consumers complained of inadequate care when one delivered at night,

“there is minimal attention given to the mothers by the health providers as they usually wait and come when the baby is almost out”

This could be addressed by a regular quality assurance mechanism. They mentioned that sometimes incentives packages run out**,** and mothers go away without. Some customers were unaware that registering and having the m-card enabled one to deliver in another county. Others had been turned away from certain private hospitals. Consumers felt that the period of free care was too short and needed to be lengthened. Such issues needed to be clarified.

Respondents cautioned against over reliance on *Linda Mama* as.

“Without it everything can come to a standstill, if it stops then the indicators will go down again” exclaimed a manager.

There was increasing dependency among mothers those were no longer putting enough effort in preparing for their babies’ deliveries because they knew that they would be given everything during delivery. High expectations appeared to be increasing and needed to be handled with care. High expectations seemed to be expressed also by managers and service providers. Some managers were of the view that the basic package covered was not comprehensive enough since it did not cater for X rays, routine ultra sounds to be done during ANC as these could not be covered in the Kenya shillings 600 reimbursed. In their view, lack of these diagnostic services affected adequacy of timely diagnosis of some conditions during ANC.

Not all health facilities were implementing the initiative, for example some dispensaries did not operate at night and hardly conducted deliveries, which respondents thought was a is a missed opportunity to provide easily accessible services to local populations. In addition, many facilities did not work during weekends making hospitals to be overwhelmed during weekends. Respondents also mentioned inadequate sensitization about the initiative at the grassroots leading to a knowledge gap among mothers regarding the initiative and its importance. Community engagement element was inadequate in all the three counties.

## Discussion

### Emerging concepts, principles, pathways for UHC

In this section we distil, discuss and reflect on the learning’s from these key concepts, principles and pathways of successful health interventions from the findings, and recommend that they be included in the policy guidelines for the design and implementation the universal health coverage currently being piloted in four counties in Kenya. The concepts, principles and pathways include: involvement, partnership, community engagement, inclusive participation, equity, basic essential care package, sustainability, output-based incentives and enhanced capacity for quality service provision, easing financial burden for consumers with autonomy and respect, and pathways for attitude change. Several studies [18, 21–24] have been conducted on the free maternity policy in Kenya. The main focus of our study was to find out the views and perspectives of the *Linda mama* implementers and consumers using qualitative research methods. We sought to understand from their view points whether the initiative had influenced utilisation of essential maternal health services in Kenya and what were the mechanisms and pathways involved.

### Awareness of, involvement, partnership engagement and inclusive participation

#### Awareness and involvement

Those in the health system and at the health facilities such as managers and service providers were well aware of the initiative and were involved in it. However the consumers were neither aware nor involved to the same level as managers and service providers. Only a small fraction among ante-natal attendees had heard of the initiative and fewer still had actually enrolled in it. Post-natal mothers were more aware and more of them had registered, indicating inadequate communication of the initiative to potential consumers. This finding is support by the findings of Orangi and her collegues who reported lack of proper channels of communication leading to lack of awareness of the initiative, and a lack of clarity on the benefit package among both the implementers and beneficiaries [18]. There appeared to be a knowledge and awareness gap among those that had not reached the health facilities. Participation in “*Linda Mama*” seemed to be more prominent among managers and service providers, who appeared to have been better reached by policy dissemination.

#### Partnership and community engagement

The Linda mama initiative was a partnership led by the Kenya Government, the Ministry of Health, and NHIF. Each of these partners plaid key roles in resourcing and implementing the scheme. Various agencies were mentioned to different extents in different counties, but they were all supporting the Government initiative. The aspect of community engagement in the initiative was not brought out clearly by the respondents, although some mentioned household visits by the CHVs to sensitize households, and output based incentives for referring mothers. Respondents highlighted the need for a more robust sensitization through greater inclusive community participation in the design and implementation of the initiative.

The evidence on the cost-effectiveness of community-based workers from literature is overwhelming [25]. It is community-based workers that can reach every household, and every woman to ensure universal coverage by “Linda Mama”, reinforced by media and social media messaging. Routine household visits by the CHVs to sensitize them and output-based incentives for referring mothers, led to good performance in villages where the CHVs were active in sensitizing and linking households to health facilities. Community engagement should be core to the initiative, to ensure that no one is left behind. The community based workers should therefore be trained, and supported because they are the ones who are directly in touch with households, individuals, and groups and have a key role to play in advocacy, sensitization, mobilization of community members and resources. They should register all households into a digital data base. Data collected by community health workers has been shown to be adequate in reliability and validity [26].

#### Inclusive participation

Inclusive community participation is recognized by researchers as critical to health interventions requiring trust in health service providers and promoting care seeking [27], bringing all perspectives to the discussion through local structures, networks, and leaders grounded in community trust with sustainable structures to reinforce resilience. It is key in ensuring that every member of the community affected by an intervention such as “Linda Mama” initiative is equitably represented at all levels making decisions, influencing actions, quality, entitlements ensuring they are fair according to their needs, aspirations, and rights, enabled through formal and traditional networks, leadership systems and structures applied to the health system. Although Community Health Volunteers (CHVs) were mentioned, without a recognized community base and supervisory support their role in the scheme would be substantially weakened. The key point is that the community ought not to be a passive actor in the initiative as they seemed to be in the assessment reported in this article. The community should play an active role in identifying the issues to be addressed and giving direction as to the ways and means in resolving issues.

#### Establish the initiative on social capital

Social capital implies investing in relationships to enable reciprocal support and solidarity within and between communities, community health units in community health strategy and social groups provide a strong foundation for “Linda Mama” initiative to ensure realistic expectations and sustainability. Social capital is based on the fabric of trust, shared values, shared mental models, joint problem perceptions and relational skills based on underlying principles of interdependence and understanding that allows diverse participants to work together towards collective outcomes and common goals [28], and ensure that people have access to social structures, and resources vital to livelihoods. Involving elements of social capital in policy dissemination and implementation have been shown to improve uptake and effectiveness of health interventions. Wisner & Kelman and Noel et al. [29, 30] describes these concepts and the association of social capital with health outcomes.

### Ensuring Equity in recruitment and access to care

The managers and providers of services displayed profound awareness of how requiring identification Cards (IDs), and telephone numbers had the potential to undermine equity by excluding those in greater need of care such as under-age pregnant adolescents and other disadvantaged groups who may not have IDs or phones. The initiative provided many options and alternatives to minimize the potential equity impact. However requiring that customers bring photocopies of IDs could open up room towards exploitation of the disadvantaged. These should be provided for at the service delivery, registration point to make sure that no one could be excluded by lack of cash when needing to register for care. Asking women to pay for photocopying introduces a cost that could be a barrier to the most vulnerable. Same findings have been reported by other researchers. Orangi et al.in their study reports that in some cases out of pocket payments were still being levied to beneficiaries of the *Linda Mama* programme [18].Cost has been shown to be a barrier to accessing care even in high income settings [31]. The registration number, the notification, written in their ANC booklets, enabled them to obtain maternity services and postnatal care anywhere in Kenya. Providing a computer, photocopier, and internet services for registration and data management for all may be useful in reducing costs for mothers and thus enhance access to care. In the context of scarcity, one has to make choices, and may have to for-go items whose cost-effectiveness have not been shown.

### The basic essential sustainable quality care package, avoiding dependency

The managers, and service providers described an impressive range of an integrated comprehensive service package, as defined in the Kenyan Essential Care Package, Strategic plan II [32]. This care package was available to the pregnant women and their children free of charge, supported by a referral system. Several studies have shown that costs are a barrier to access of maternal health services in Kenya with majority of the poor delivering at home [33, 34]. User fee removal for maternal health has been shown to increase health facility delivery among the poor in Kenya [35]. Although our study was purely qualitative, our findings add to the evidence by demonstrating that that the initiative substantially increased utilization of services. Researchers have reported similar trends in Burkina Faso, Mali, Benin and Malawi [36–38].

Although some managers and service providers felt that *Linda Mama* with material incentives may be creating dependency, and high expectations for a level of support that the Government may not be able to sustain in UHC. It is critical that the cost of an initiative is fixed at a level that the Government and community can afford long term [39], which is key to primary health care, according to Alma Atta Declaration [40]. Other mechanism for providing such material support based on community solidarity should be considered.

As it is, consumers complained that these **i**ncentive packages often ran out**,** and some mothers had to go away without them, feeling deprived and treated unfairly. Since consumers are maximisers of satisfaction, it is impossible to establish incentives that would be sufficient. From consumer respondents the demand for more items to be included appeared unlimited in the phase of obviously limited resources. High and growing expectations was also displayed among managers, and service providers with regard to what should be included in the Linda mama package, asking for X ray, ultra sound examinations that may not currently be included for reimbursement, and in their view hindering adequacy of diagnosis during ANC. What must be included in the package should be based on a cost-effectiveness assessment, in keeping with the spirit of essential care package for health. Maternal and new-born packs and additional diagnostics at the primary care level may be moving the attention of the partners away from the core service provision issues that they should be focusing on, in terms of value addition and cost per unit of health improvement, thus undermining sustainability.

***Sustainability*** concerns the maintenance of positive change, through a robust and enduring infrastructure, human capital, resources, and governance systems that continue to generate benefits for the population without depleting the environment, or other resources. Central to it is implementing partners ensuring that the per capita cost of the initiative such as *Linda Mama* is within a range that the state can sustain beyond the pilot period as described by Newbrander [41], based on meaningful allocation of state resources to the health sector and effective community engagement.

### Mutual health system provider and consumer benefits of *Linda Mama*

The genius of the initiative is its ability to benefit the health system, the providers and managers on the one hand and the individual consumers, households and communities on the other hand at the same time. The benefit was all round, both facility and the clients benefitted. It had improved quality of care for the mother and baby, by reducing the cost of referring mothers for specialized services such as ultra-sound services. It improved structure, process and outcomes. The free MCH services eased life for mothers particularly for those who did not have money at the time of delivery by reducing the cost of seeking care to a minimum at the time of need. While at the same time enabling the health system and its managers and providers to ensure availability of the resources, infrastructure and the environment they needed to provide services to the satisfaction of consumers through an output-based approach to financing. Maternity and MCH services improved as a result of the funds received by health facilities. “Linda Mama” reimbursements helped to purchase drug and reduced workload in the facility by hiring extra handsThe main challenge was the delay in disbursement of funds to the facilities. As a model for UHC implementation, these challenges must be urgently addressed. Suggestions included direct reimbursement to the health facilities and not through the County Health management system. Gaps identified by the consumers such the process of care at night being inadequate should be urgently addressed by applying a quality assurance and continuous improvement strategy at all levels of care capturing the structures, processes and outcomes of care at all levels and in all participating health facilities, to entrench a culture of continuous quality improvement. It eased family economic burden on health, reduced costs for the consumer to the minimum, while not undermining their autonomy and choice for the service provider, which is an ethical principle that must be included in the UHC initiative, by enhancing the social justice dimension, central to health interventions, placing priority on those most vulnerable is a hallmark of social justice [42]. The approach makes good economic sense as described by Frenk and Ferranti [43].

## Conclusion

The *Linda Mama* initiative seems to have influenced attitudes on health facility delivery through three pathways to be considered in the implementation of UHC by the Kenyan Government:

1. Partnership among key stakeholders, which include the National Government, National Health Insurance Fund, and County Governments, but highlighting the need for enhanced partnership with the communities
2. Enhancing the capacity of the health facilities to deliver a high quality comprehensive, essential care package through output-based financing
3. Easing economic burden of health care for households to ensure that everyone could access care while ensuring their autonomy in choosing the service provider of their choice. Thus *Linda Mama* initiative enabled the improvement of structures for care provision, processes of care provision and access to services and hence coverage. It enhanced health facility capacity to handle the patients’ needs and thus regained confidence among consumers, during pregnancy, childbirth, to the expiry date, convinced that delivery at the health facility is much safer as complications can be effectively handled. There was substantial quality improvement, environment, equipment, doctors and essential commodities were available, processes and outcomes. It appears to have transformed the attitude of consumers concerning going to health facilities for skilled delivery. To sustain this change, all health facilities should be included.

## Data Availability

The datasets used and/or analyzed during the current study are available from the corresponding author on request.

## Abbreviations

ANC: Antenatal care
CAC: Comprehensive Abortion Care
CHV: Community Health Volunteer
HIV: Human Immune Virus
KMET: Kenya Medical Emergency Treatment
MCH: Maternal and Child Health
MOH: Ministry of Health
NHIF: National Health Insurance Fund
PNC: Post-Natal Care
SDG: Sustainable Development Goal
TBA: Traditional Birth Attendant
UHC: Universal Health Coverage
UNICEF: United Nations Children Fund
UNDP: United Nation Development Programme
WB: World Bank
WHO: World Health Organization
